# Comparison of the sensitivity of the EULAR / ACR 2019 and SLICC 2012 classification criteria in a Colombian population with systemic lupus erythematosus

**DOI:** 10.1016/j.jtauto.2021.100133

**Published:** 2021-11-10

**Authors:** Diana Guavita-Navarro, Laura Gallego-Cardona, Ana María Arredondo, Hector Cubides, Jairo Cajamarca-Barón, Claudia Ibáñez, Alejandro Escobar, Adriana Rojas-Villarraga

**Affiliations:** a Department of Rheumatology, Hospital de San José, Fundación Universitaria de Ciencias de la Salud-FUCS, Bogotá, 111221, Colombia; b Research Division, Fundación Universitaria de Ciencias de la Salud-FUCS, Bogotá, 111221, Colombia

**Keywords:** Systemic lupus erythematosus, Prevalence, Criteria, European league against rheumatism (EULAR), American College of rheumatology (ACR), Systemic lupus international collaborating clinics, American College of rheumatology (SLICC)

## Abstract

**Background:**

*/Objective*: Systemic lupus erythematosus (SLE) is a multisystem autoimmune disease with a wide range of clinical manifestations. The latest classification criteria, EULAR/ACR 2019, have not been validated in a Latin American population of Amerindian ancestry. The objective of this study is to compare the sensitivity of the EULAR/ACR 2019 and SLICC 2012 classification criteria in a group of SLE patients with the above ancestry.

**Methods:**

A cross-sectional study was done. Data were obtained from the review of medical records of patients who met the inclusion criteria. The overall sensitivity of the criteria was calculated and compared to each other using the McNemar test.

**Results:**

146 medical records of patients from two referral centers were included. There were no differences in the sensitivity of the EULAR/ACR and SLICC 2012 criteria (84.9% versus 85.6% p = 0.79) nor were differences found when the groups based on disease duration were compared: less than 5 years (91.0% versus 92.5% p = 0.70), between 5 and 10 years (82.8% versus 82.8% p = 1), and 10 years or more (76.7% versus 76.7% p = 1). However, SLICC 2012 criteria was found to better classify patients with a less than 5-year disease duration compared to those with 10-year duration or more (92.5% versus 76.4% p = 0.024).

**Conclusions:**

There are no statistically significant differences between the EULAR/ACR and SLICC 2012 criteria in the population studied. Nor were differences found when evaluating them by age at diagnosis and duration of the disease except when the group with less than 5 years of duration was compared to those with 10 years or more using the SLICC 2012 criteria.

## Highlights

• There are no differences when the sensitivity of the two sets of criteria are compared.
• SLICC criteria better classify patients with a less than 5-year disease duration compared to those with 10-year or more.
• There are no differences between the two sets of criteria for classifying patients with childhood-onset lupus.

## 1 Introduction

Systemic lupus erythematosus (SLE) is an autoimmune disease that is frequently multisystemic. However, it can sometimes involve a single organ. It predominantly affects women with a peak onset before the age of 45 and an incidence that in North America ranges from 72.8 to 102 cases per 100,000 people per year although it is twice as high in Hispanics. In Colombia, the prevalence has been calculated at 0.05% which is approximately 91.9/100,000 people with a female to male ratio of 7.9:1 and a peak incidence between ages 45 and 49 [[1], [2], [3], [4], [5]]. The clinical presentation is variable and compromises any organ or system mildly or severely [6].

Historically, classification criteria such as the 1982 American College of Rheumatology (ACR) that have a sensitivity and specificity of 96% and were created by nine experts from the American Rheumatism Association (ARA) have been used [7]. Given the high heterogeneity of cutaneous lupus and the possibility that patients might be classified as SLE with only mucocutaneous manifestations and the absence of renal histology, it was necessary to develop new criteria [7].

The Systemic Lupus International Collaborating Clinics (SLICC) 2012 criteria [8] were then developed. These held to the original idea of the ACR 1982 criteria by adding new items such as the inclusion of a clinical and an immunological criteria, low complement, and direct positive Coombs test in the absence of hemolysis. Furthermore, renal histology in combination with antinuclear antibodies (ANA) or anti-double-stranded DNA antibodies (ds-antiDNA) was accepted as a sufficient classification criterion. The hematological criterion was further subdivided into hemolytic anemia, lymphopenia, and thrombocytopenia. Subacute cutaneous lupus, bullous lupus, maculopapular rash, and chronic cutaneous lupus were included in the cutaneous compromise, and photosensitivity was removed. There were several additions from the neurological domain such as mononeuritis multiplex, transverse myelitis, cranial or peripheral neuropathy, and acute confusional state [8]. The sensitivity achieved by these criteria was 97% which was higher with respect to the ACR 82 although the specificity, 84%, was compromised [9].

In 2019, new classification criteria came to light which increased the sensitivity of its predecessor, ACR 1997 [10], while preserving the specificity. When the criteria were validated, a sensitivity of 96.1% and a specificity of 93.4% were achieved thus surpassing ACR 1997 (82.8% and 93.4% respectively). There were 3 main modifications to these criteria. The first modification, the Antinuclear antibodies (ANAs), was included as an entry criterion. The second one was to give the criteria relative weight, and the third only allows them to be applied in the absence of another more likely explanation [7,8]. It is noteworthy that out of the 696 patients with SLE included in the study to develop new criteria only 73 were Hispanic, a population that has a higher risk of kidney involvement and a worse prognosis [11].

As expected, studies have been done comparing these new criteria with the old ones. One of the first publications is that of Dahlstrom [12],which compares the 2012 SLICC criteria with those currently proposed and concludes that they are similar with respect to sensitivity and specificity. Another study, which included 293 patients diagnosed with SLE [13], showed similar results in terms of the absence of differences between European League Against Rheumatism (EULAR)/ACR 2019, SLICC 2012, and ACR 1997. It should be noted that there have also been studies of the pediatric population such as the one done by Batu et al. [14] who evaluated the 3 criteria included in the previous study and documented a sensitivity of 68.7%, 95.4%, and 91.6% and a specificity of 94.8%, 89.7%, and 88.5% respectively. They concluded that SLICC and ACR 1997 perform better in sensitivity and specificity compared to EULAR/ACR 2019 in this population. At the Latin American level, the Latin American Lupus Study Group (GLADEL) compared the new criteria to ACR 1997 and SLICC 2012 [15]. The sensitivity calculated for EULAR/ACR 2019 compared to ACR 1997 criteria, which was taken as the gold standard, was 91.3%. In addition, it was determined that it is possible to detect patients at earlier stages of the disease using the current criteria. However, no studies published in Colombia and found so far have been similar to this one. A few have been done on populations that include those with Amerindian ancestry or polyautoimmunity. This is important because the two conditions – ancestry and polyautoimmunity – are important in the context of the autoimmune tautology [16,17].

The objective of this study is to compare the sensitivity of the SLE EULAR/ACR 2019 and SLICC 2012 classification criteria using a Latin American population with Amerindian ancestry from two referral centers in Bogota, Colombia.

## 2 Methods

### 2.1 Study design and data collection

A cross-sectional study was done. Data were obtained from the consecutive review of medical records of patients evaluated between 2016 and 2019 in two referral centers and were recorded anonymously in an electronic collection format. The variables collected included age, sociodemographic data, duration of the disease, age of onset, presence or absence of polyautoimmunity, comorbidities, treatments, autoimmune profile, and all those related to the two sets of criteria (2019 and 2012).

### 2.2 Study population

Two investigators did an independent review of the medical records of all patients who met the following inclusion criteria: a) diagnosis of systemic lupus erythematosus by the treating physician with International Classification of Diseases-10 codes ranging from M329, N040 to N084, from N178 to N189 and N19X recorded in the clinical history at the hospital and outpatient setting; b) availability of all data from the clinical history including the various clinical and paraclinical variables to corroborate both sets of criteria. Those patients under 18 years of age or those with only cutaneous or drug-induced lupus were excluded.

This work guarantees the confidentiality of the participants' data, adheres to the principles of the Declaration of Helsinki, and was approved by the research ethics committees of the participating centers (Ethics Committee on research with human beings of the Fundacion Hospital Infantil Universitario de San Jose Act number 70) and by the ethics committee for research on human beings HSJ-FUCS/CEISH Act number 576. The personal information was kept anonymous, and a specific code was generated for each subject during the database development and analysis.

### 2.3 Statistical analysis

The data were exported from the digital format to a database in the Excel ® program and later analyzed using the statistical package STATA version 15 ®. The frequency of SLE was calculated based on the EULAR/ACR 2019 and SLICC 2012 criteria by means of the number of patients who met the classification criteria. A descriptive analysis of the variables of interest was done using measurements of central tendency and dispersion for the quantitative variables and using absolute and relative frequencies for the qualitative variables. The age variable was summarized using the median and interquartile range. The normality of the data was assessed using the Shapiro Wilk test. Fisher's exact test was used to evaluate the difference in proportions. The overall sensitivity of the criteria was calculated and compared to each other using the McNemar test. The level of statistical significance was established with a p < 0.05.

## 3 Results

One hundred and forty six patients with a diagnosis of SLE from 2 reference centers in Bogotá, Colombia were included. The median age of the cohort was 36 (interquartile range 26–51), women predominated in 82.8% of the cases, and the duration of the disease was less than 5 years in 46.2%. Regarding treatment, 98.6% and 87.6% received antimalarial and corticosteroids respectively. One hundred thirty five (92.4%) patients had positive ANAs, and the most common pattern reported was homogeneous followed by granular. Patterns or dilutions were not reported in the medical history of five patients. Of these, only one met the SLICC 2012 criteria, and the others did not meet any of the sets of criteria. In addition, 6 patients were found with negative ANAS, 5 met SLICC 2012 criteria and none met the EULAR/ACR 2019 criteria (it is an entry criterion). Regarding comorbidities, hypertension and smoking stand out. The patients provided by Hospital San José from the outpatient area were classified 99 (90%) by EULAR/ACR 2019 and 94 (85.4%) by SLICC 2012 criteria. Of the patients included from the hospitalization area of the Fundación Hospital Infantil Universitario de San José, 25 (69%) met the EULAR/ACR 2019 criteria and 31 (86.1%) the SLICC 2012 criteria. Of the total population studied, 90.4% met at least one of the qualifying criteria for SLE, 125 (85.6%) met the 2012 SLICC criteria, and 124 (84.9%) met the EULAR/ACR 2019 criteria. Of the 125 patients who met the 2012 SLICC criteria, 4 patients did so due to biopsy-proven lupus nephritis and the presence of positive ANAS [one of them with additional positive Anti-double stranded DNA (Anti-dsDNA)] without meeting other additional criteria. The demographic characteristics of the population studied are presented in Table 1. Table 2 and Table 3 show the clinical characteristics that make up any of the two sets of criteria.

**Table 1:** Description of the population studied.

Characteristics	n(%) 146 (100%)
Year (yr)	36 (median)
Age at diagnosis	n/145
Over 16 years of age	123 (84.83)
Under 16 years of age	22 (15.17)
Duration of illness	n/145
<5 years	67 (46.2)
5–10 years	35 (24.1)
>10 years	43 (29.66)
Gender	n/146
Female	120 (82.2)
Male	26 (17.8)
Occupation	n/146
Manual trades	40 (27.4)
Home	29 (19.86)
No data	27 (18.4)
Student	26 (17.81)
Office and administrative trades	17 (11.64)
Pensioner	7 (4.76)
Social Security	n/146
Contributory	72 (49.32)
Beneficiary	48 (32.88)
No data	15 (10.27)
Subsidized	9 (6.16)
Special regime	2 (1.37)
Institution	
San Jose Hospital (Outpatient setting).	110
Fundación Hospital Infantil Universitario De San José de Bogotá (Hospitalizad).	36
Background	
Arterial hypertension	28/142 (19.72)
Smoking	24/134 (17.91)
Infections	10/141 (7.09)
Osteoporosis	7/129 (5.43)
Diabetes Mellitus	6/137 (4.38)
Previous or current cancer	6/134 (4.48)
Alcohol	5/137 (3.65)
Dyslipidemia	4/125 (3.20)
Treatment	
Corticosteroids	128/146 (87.6)
Chloroquine	99/144 (68.75)
Azathioprine	75/143 (51.45)
Hydroxychloroquine	45/142 (31.6)
Cyclophosphamide	36/143 (25.17)
Mycophenolate	30/145 (20.69)
Methotrexate	22/144 (15.28)
Biological therapy	11/139 (7.91)
ANAS value	n/130
1/320	39 (30.0)
1/1280	34 (26.15)
1/160	24 (18.46)
1/640	21 (16.15)
1/80	11 (8.46)

**Table 2:** Compliance with SLICC 2012 Criteria in the population studied.

Criteria	N (%)
N Total	146
Meets SLICC 2012 criteria	125 (85,62)
**Acute cutaneous lupus**	45 (30.82)
Photosensitive lupus rash	*34/139 (24.46)*
Lupus malar rash	*19/138 (13.77)*
Toxic epidermal necrosis	*3/129 (2.33)*
Subacute cutaneous lupus	*3/134 (2.24)*
Maculopapular lupus rash	*1/134 (0.75)*
**Chronic cutaneous lupus**	10 (6.85%)
Classic discoid rash	9/143 (6.29)
Lupus panniculitis	1/138 (0.72)
Discoid lupus/lichen planus overlap	1/137 (0.73)
**Oral or nasal ulcers**	32/135 (23.7)
**Non-scarring alopecia**	40/137 (29.2)
**Synovitis**	93/143 (65.03)
**Serositis**	33 (22.6)
Pleural effusion	22/140 (15.71)
Pericardial effusion	20/140 (14.29)
Pleurisy	8/140 (5.71)
Pericardial pain	7/140 (5.0)
Acute pericarditis	6/141 (4.26)
Pleural rub	5/133 (3.76)
Pericardial rub	5/133 (3.76)
**Renal**	56/146 (38.35)
24-h urine proteinuria	49/140 (35.0)
Urine protein–to-creatinine ratio	27/126 (21.4)
Red blood cell casts	18/134 (13.4)
**Neurological**	23/146 (15.75)
Seizures	9/141 (6.38)
Cranial or peripheral neuropathy	9/134 (6.72)
Psychosis	6/142 (4.23)
Acute confusional state	4 (2.84)
Myelitis	2/141 (1.42)
Mononeuritis Multiple	1/142 (0.70)
**Hemolytic anemia**	37/142 (26.0)
**Lymphopenia**	48/146 (32.88)
Leukopenia	35/138 (25.36)
**Thrombocytopenia**	39/136 (28.6)
**Immunological criteria**	
**Anti dsDNA**	74/131 (56.49)
**Anti SM**	47/119 (39.50)
**Antiphospholipid Antibodies**	44/146 (30.1)
LA	30/101 (29.7)
IGG aCL	16/103 (15.53)
IGM aCL	16/100 (16.0)
IGA aCL	6/46 (13.04)
IGM B2GPI	11/83 (13.25)
IGG B2GPI	10/83 (12.05)
**Low complement**	100 (68.5)
Low C4	87/135 (64.44)
Low C3	86/136 (63.24)
Low CH50	1/26 (3.85)
**Positive direct Coombs' test**	28/65 (43.08)

ANA: Anti-nuclear antibody, anti-dsDNA: anti-double-stranded deoxyribonucleic acid, anti SM: anti-Smith, aCL. Anti-Cardiolipin, B2GPI: anti-β2-glycoprotein I, CH50: total complement activity, LA: Lupus anticoagulant, SLICC: Systemic Lupus International Collaborating Clinics.

**Table 3:** Compliance with EULAR/ACR 2019 Criteria in the population studied.

Criteria	N (%)
N Total	146 (100)
EULAR/ACR 2019	124 (84**.**93)
**Fever**	8/140 (5.71)
**Mucocutaneous**	67 (45.9)
**Hematological**	84 (57.5)
Thrombocytopenia	39/136 (28.68)
Autoimmune hemolysis	37/142 (26.06)
Leukopenia	35/138 (25.3)
**Neuropsychiatric**	16 (10.9)
Seizures	9/141 (6.38)
Psychosis	6/142 (4.23)
Delirium	4/141 (2.84)
**Mucocutaneous**	67 (45.9)
Non-scarring alopecia	40/137 (29.2)
Oral ulcers	32/135 (23.7)
Subacute cutaneous lupus or discoid lupus	12/146 (8.2)
Acute cutaneous lupus	7/131 (5.34)
**Serosal**	31 (21.2)
Acute pericarditis	6/141 (4.26)
Pleural effusion or pericardial effusion	31/140 (22.14)
**Musculoskeletal**	93 (63.7)
**Renal**	56 (38.35)
Proteinuria > 0.5 g/24h	49/140 (35)
Renal biopsy Class III or IV kidney	22/128 (17.19%)
Renal biopsy Class II or V kidney	17/129 (13.18%)
**Immunological domains and criteria**	
**Antiphospholipid antibodies**	45/146 (30.8)
LA	30/101 (20.5%)
IGG aCL	16/103 (15.53%)
IGM aCL	16/100 (16.0%)
IGA aCL	6/46 (13.04%)
IGM B2GPI	11/83 (13.25%)
IGG B2GPI	10/83 (12.05%)
IGA B2GPI	3/41 (7.32%)
**Complement**	99 (67.8)
Low C3 OR low C4	26/146 (17.81)
Low C3 AND low C4	73/146 (50.0)
**SLE-specific antibodies**	91 (62.3)
Anti dsDNA	74/131(56.49%)
AntiSM	47/119 (39.50%)

ACR 2019.2019 Classification of the European League Against Rheumatism and the American College of Rheumatology for the diagnosis of systemic lupus erythematosus, ANA: Antinuclear antibody, anti-dsDNA: anti-double-stranded deoxyribonucleic acid, anti-SM: anti-Smith, aCL. Anti-Cardiolipin, B2GPI: anti-β2-glycoprotein I, CH50: total complement activity, LA: Lupus anticoagulant.

The main extra-criteria manifestation found was the Raynaud phenomenon. Polyautoimmunity was found in 57 patients (39%). Antiphospholipid antibody syndrome was the most frequent followed by rheumatoid arthritis, and Sjögren's syndrome. Multiple autoimmune syndrome presented in 10 patients with 3 autoimmune diseases including SLE. Additionally, the most frequent non-specific antibodies were anti-RO, anti-LA, and anti-RNP. At the same time, a family history of autoimmunity was found in 17.8% of the sample.

No differences were found when comparing the sensitivity of the EULAR/ACR 2019 and SLICC 2012 criteria in the study population (84.9% vs. 85.6% p = 0.79). Nor were differences found in the number of patients classified by EULAR/ACR 2019 when they were 16 years old or younger compared to those who were 17 years old or older at the age of diagnosis (86.3% vs. 84.5% p = 0.56). The same situation occurred when the proportions were analyzed by SLICC 2012 (90.9% Vs 84.5% p = 0.34).

When sensitivity was analyzed by comparing the two sets of criteria in the age groups at diagnosis, the results in the age 16 or under group were 86.3% and 90.9% for EULAR/ACR 2019 and SLICC 2012 respectively (p = 0.56). They were similar in the age 17 and above group where there were no significant differences between the two criteria (84.5% Vs 84.5% p = 1). The specificity was not analyzed despite its clinical importance because this study did not include a population divided between sick and healthy which is needed to calculate specificity.

The percentage of individuals who were classified by EULAR/ACR 2019 criteria based on the duration of the disease was 91.04% for less than 5 years, 82.8% for between 6 and 10 years and 76.7% for more than 10 years from onset (p = 0.104). Likewise, for SLICC 2012, the calculated proportions were 92.5%, 82.8%, and 76.7% for less than 5 years, between 6 and 10 years, and more than 10 years from onset (p = 0.061) respectively. A trend was documented in which more patients with a less than 5-year duration of lupus were diagnosed by SLICC 2012 criteria. Based on this same set of criteria, an analysis was done of the above subgroup compared to the group with a 10-year duration or more which found statistically significant differences [67 (92.5%) vs 33 (76.4%); p = 0.024]. For the EULAR/ACR 2019 criteria set, the differences between the same two groups showed a similar trend but one that was not statistically significant (p = 0.052).

A comparison was done in each disease duration group of the sensitivity of the two criteria which showed no differences between any of the 3 groups (See Table 4).

**Table 4:** Comparison of the criteria based on the categories of disease duration.

Duration of SLE after diagnosis	EULAR/ACR criteria sensitivity%	Sensitivity of SLICC criteria 2012%	P^a^
Any duration	84.9%	85.6%	0.79
Less than 5 years	91.0%	92.5%	0.70
5–10 years	82.8%	82.8%	1
More than 10 years	76.7%	76.7%	1

a McNemar Test.

Fourteen patients did not meet either of the two criteria. Twelve were female, four did not have complete ANA data, three participants had acute skin involvement and, of these, one case was associated with a joint component. Four patients had systemic compromise without an immunological component. One of the patients had a renal biopsy compatible with class III lupus nephropathy associated with complement consumption but without the presence of ANAs or specific antibodies.

## 4 Discussion

This study demonstrated that there is no statistically significant difference between the EULAR/ACR 2019 and SLICC 2012 criteria in a group of patients that represent a real-life clinical practice in the Colombian population (outpatient and hospital). Nor were differences found when evaluating them by age at diagnosis or duration of the disease except when the group with less than 5 years of duration was compared to those with a greater than 10-year duration using the SLICC 2012 criteria.

One of the most representative studies done in Latin America is that of Pons-Estel, of the Latin American Lupus Study Group (GLADEL) [15] where they did a comparative analysis of the EULAR/ACR 2019 criteria and the ACR 97 in a cohort in which Caucasians and mestizos predominated. As is typical in autoimmune diseases in general and our study in particular, more women participated with a slightly lower mean age than in our cohort (29.8 vs. 36 years). The sensitivity found for the new criteria was 91.3%, which contrasts with the sensitivity found, 84.9%, in our study. Another cohort with interesting results is LUMINA (Lupus in Minorities: Nature Vs Nurture) [18] in which Spanish-American patients are also analyzed. These were in line with the GLADEL group in which patients who meet EULAR/ACR 2019 criteria belong to a subgroup with more severe disease. In addition, a few patients included in the study achieved the main objective of being classified early as originally proposed when the criteria were developed. Another Latin American study which compared the SLICC 2012 criteria with the ACR97 showed that the former are more sensitive than the latter [19].

In patients with a less than 5-year duration of SLE, the SLICC 2012 was better at classifying them unlike the EULAR/ACR 2019 criteria which showed only a trend. These data coincide with the study by Ines et al. [20] where the SLICC 2012 were more sensitive in classifying patients (p < 0.0001) than the ACR 97 criteria. Adamichou et al. [21] did a retrospective observational study comparing the EULAR/ACR 2019, SLICC 2012, and ACR 97 criteria in patients with early disease (48 months). The study demonstrated that the first two were more sensitive than ACR 97 to the population under study. Lobo Prat [22] also compared EULAR/ACR 2019 and SLICC 2012 without documenting differences in sensitivity with respect to patients with long disease duration and had results similar to those in studies by Vrancianu [23] and Duarte-Garcia [24]. The Johnson et al. [25] study included patients with Hispanic ancestry and got results that contrast with ours given that, for them, the EULAR/ACR 2019 criteria were better on patients with early disease in terms of sensitivity. Others have gone further and documented that a score greater than 20 points for SLE of less than 5 years duration is related to greater disease activity, less probability of reaching remission, and high immunosuppression [26].

Childhood-onset lupus, unlike adult-onset, is more aggressive with high disease activity, greater use of immunosuppressants, and accumulated damage that causes greater morbidity and mortality [27]. Despite these important differences, there are no specific classification criteria for this population group, which is why, over time, the same criteria have been used as in adults. Aljaberi [27],for example, concludes that the EULAR/ACR 2019 criteria are more sensitive than the ACR 97 although the specificity is similar. Regarding the 2012 SLICC, they have also been shown to be more sensitive when compared to ACR 97 [28]as corroborated in a Colombian study that included 110 pediatric patients. However, the specificity found was lower [29]. No differences in the sensitivity of the two criteria were found for the classification of these patients although a study done in Brazil by Rodríguez Fonseca et al. [30] indicates that the SLICC 2012 are better when compared with EULAR/ACR 2019 and ACR 97. However, they stated that the performance of the 2019 criteria in the youth population can be improved if they are classified using 13 points and not 10 as originally proposed. Other recent studies do not find differences between the three criteria (EULAR/ACR 2019, SLICC 2012, and ACR 97) [31]. However, this contrasts with data published by Levinsky et al. [28] who documented a better sensitivity of the EULAR/ACR 2019 with respect to the SLICC 2012 in a population with juvenile SLE.

In the present study, of the 6 patients with negative ANAs, 5 met the qualifying criteria. Even so, this information did not generate differences between the two sets. This confirms the great heterogeneity of the disease and gives way to the concept of seronegative SLE, first described in 1970, where, despite the absence of antinuclear antibodies, patients presented systemic clinical manifestations of the disease and therefore, the diagnosis was always based on the judgment of the treating physician rather than just the criteria. Over time, the behavior of this population will be evaluated in Latin American cohorts [[32], [33], [34]].

In the present cohort, a high percentage (39%) presented polyautoimmunity. This correlates with the literature where a prevalence of up to 41% has been calculated, and autoimmune thyroiditis, Sjögren's syndrome, and antiphospholipid antibody syndrome have been the most frequently associated diseases. This has had a negative impact on the course of the disease given that they cause more severe manifestations [35,36]. However, at the time the criteria were developed, patients with other concomitant autoimmune diseases were excluded. Our results and those already reported in the literature encourage us to continue evaluating the performance of the criteria in this group in the Latin American population [37].

In our study, joint compromise was the most frequent in the two sets of criteria, followed by hematological for SLICC 2012 and mucocutaneous for EULAR/ACR 2019. Renal involvement occurred in the same proportion in the two groups. These data correlate positively with the publication by Lobo Prat, where significant associations were found between fulfillment of the criteria and the presence of arthritis and lupus nephritis [22]. Furthermore, some studies have evaluated the current criteria as a possible prognostic tool. The results of the study by Carneiro et al. suggested that the high scores in the current criteria are associated with a high index of organ damage, especially kidney [38]. Another study predicts hospitalizations within two years of a score greater than 19 [39].

Our study has some limitations. First, in the absence of a gold standard, the diagnosis was based on the perception of the treating rheumatologist. Second, when data was obtained from medical records, some of it was missing and that did not allow clinical or immunological variables to be obtained. This could explain why, in our cohort, which corresponds to real-life patients, the sensitivity of the criteria is less than that calculated in the validation study. Consequently, the number of patients with CH50 measurement, direct Coombs, serology for syphilis, and antiphospholipid antibodies is low.

The intention of this study was not to find or compare specificity which may be done better with the ACR 1997 criteria [8]. Likewise, the degree of activity or accumulated damage in our population was not evaluated through any composite index as has been done in other populations. This could explain the differences found when the two groups – those with 5-year and those with 10-year disease duration – were compared using the SLICC 2012 criteria classification. However, the damage mentioned above was not measured in the present study.

In summary, the EULAR/ACR 2019 and SLICC 2012 criteria do not differ in terms of sensitivity in our population or when they are evaluated by age group or time of diagnosis. Although the classification criteria are tools that seek to homogenize the patients included in clinical studies, they are frequently used in clinical practice as a diagnostic aid. However, it is possible that they are being used improperly, especially in the Colombian population since, according to the data from the GLADEL cohort, Latin American people have a higher rate of early renal involvement [11]. That is why our study, the first one of its kind in our country, is important. It is also our opinion that similar studies should be done with a larger population sample to obtain tools that would make it possible to use the criteria safely in real life clinical practice.

## Submission declaration and verification

This study has not been published previously and is not under consideration for publication elsewhere. All authors and responsible authorities approved its publication. If accepted, it will not be published elsewhere in the same form in English or in any other language.

## Project financing statement

This research did not receive any specific grants from funding agencies in the public, commercial, or non-profit sectors.

## Author contributions

Guavita-Navarro: Conceptualization, Methodology, Data curation, Writing, Original draft preparation, Visualization. Gallego-Cardona: Data curation, Writing, Original draft preparation, Investigation. Arredondo: Original draft preparation, Data curation, Investigation. Cajamarca-Barón: Conceptualization, Methodology, Data curation, Writing, Original draft preparation, Reviewing and Editing, Visualization. Ibáñez: Supervision, Data curation, data analysis, Reviewing and Editing, Visualization. Cubides: Data curation, Writing, Investigation. Escobar: Original draft preparation, Data curation, Investigation. Rojas-Villarraga: Conceptualization, Methodology, Data curation, Writing, Original draft preparation, Reviewing and Editing, Visualization.

## Declaration of competing interest

The authors declare that they have no known competing financial interests or personal relationships that could have appeared to influence the work reported in this paper.
